# A quest for understanding: older migrants’ wellbeing beyond integration in Sweden

**DOI:** 10.3389/fpubh.2025.1620911

**Published:** 2025-08-25

**Authors:** Anna Zhou, Åsa Larsson Ranada, Susanne Roos, Ingrid Hellström

**Affiliations:** ^1^Department of Health, Medicine and Caring Sciences, Linköping University, Linköping, Sweden; ^2^Department of Health Care Sciences, Marie Cederschiöld University, Stockholm, Sweden

**Keywords:** belonging, culture, identity negotiation, social inclusion, migration, grounded theory

## Abstract

**Purpose:**

This study investigates how older foreign-born adults in Sweden experience and navigate social connectedness as a determinant of wellbeing.

**Methods:**

Employing Glaser’s grounded theory methodology, we collected qualitative data through individual (*n* = 1) and focus group (*n* = 5) interviews with 23 participants aged 60 + representing four distinct cultural-linguistic groups: Arabic, Finnish, Spanish, and Chinese speakers.

**Results:**

The analysis identified “*a quest for understanding*” as the core category, encompassing three dimensions: (1) *wanting to be understood*, (2) *wanting to understand*, and (3) *reaching for reconciliation*. While participants shared universal needs for validation and connection, their experiences revealed tensions between aspirations and the challenges of language barriers, cultural distance, and generational differences. Notably, perceptions and experiences showed strong within-group similarities but significant between-group variations.

**Discussion:**

Framed by Nordenfelt’s concept of wellbeing as “want-equilibrium,” the findings highlight understanding as both a social need and existential pursuit. While Finnish speakers’ minority status eased integration, Arabic and Chinese speakers navigated systemic inclusion yet social exclusion. Resilience strategies—bicultural fluency, insular solidarity, or self-reliance—reflected Bourdieusian capital disparities. Wellbeing thus hinges on mutual recognition: migrants’ adaptability and Sweden’s capacity to perceive them beyond structural categories.

## Introduction

Sweden is a diverse society, with approximately 20% of its population born abroad (1). Immigration has transformed Sweden from a relatively homogeneous nation before World War II into one of Europe’s most multicultural countries today. In the 1950s and 1960s, labor migration - driven by economic expansion and labor shortages - dominated immigration patterns. From the 1970s onward, however, global conflicts and Sweden’s comparatively generous asylum policies shifted the focus toward refugee migration. Family reunification also became a significant immigration channel. Public discourse on immigration intensified after the 2015 European refugee crisis, resulting in stricter asylum policies in 2016 and a heightened emphasis on social and economic integration. As a result, today’s older foreign-born population in Sweden is highly heterogeneous, encompassing individuals with diverse migratory backgrounds. This diversity has led to wide variation in living conditions, health status, and levels of social integration (2).

Both migration and aging represent major life transitions that profoundly reshape an individual’s social capital and relational networks (3–5). Migration often disrupts long-standing social ties, compelling individuals to rebuild a sense of belonging in an unfamiliar sociocultural context (6). Aging, by contrast, is typically marked by a natural narrowing of social networks due to retirement, declining health, and the loss of peers and loved ones (7). When these transitions intersect - as in the case of older foreign-born individuals navigating migration regimes, elder care systems, and institutional structures - their social relationships and support networks may undergo complex reconfigurations within host societies (8).

Even family, traditionally regarded as the most critical source of emotional and practical support for older adults (9), may undergo structural and functional changes due to migration. Intergenerational tensions, cultural distance, and increased dependence can weaken familial ties, undermining older migrants’ autonomy and wellbeing (10, 11). These dynamics raise concerns about older migrants’ access to robust social relationships and emotional support beyond the immediate family.

Social networks, relationships, and connectedness collectively shape an individual’s lived social reality. While social networks provide the structure of interactions (who people engage with), social relationships define the quality and function of those ties (how they interact), and social connectedness reflects the resulting psychological state - whether one feels fulfilled or isolated. Drawing on Bourdieu (12), we conceptualize these elements as forms of social capital: dynamic resources embedded in networks that confer tangible or intangible benefits (e.g., support, information, opportunities). Bourdieu further highlights how social capital perpetuates inequality, as its unequal distribution allows privileged groups to convert it into economic capital (e.g., securing jobs through connections) or cultural capital (e.g., acquiring knowledge via social ties).

The significance of social connection for wellbeing is well-established across disciplines. Psychological theories- from Maslow’s hierarchy of needs (13) to Deci and Ryan’s self-determination theory (14) and Erikson’s developmental stages (15)—position belonging as fundamental to human thriving. This is especially salient in later life, as underscored by socioemotional selectivity theory (16) (prioritizing emotionally meaningful relationships) and attachment theory (17). Empirical research in public health reinforces this, linking strong social ties to better health outcomes, reduced mortality, and higher life satisfaction among older adults (18, 19). Recognizing these findings, the World Health Organization (20) has declared social connection a pillar of healthy aging, urging targeted interventions for vulnerable groups like older migrants, who face compounded risks of loneliness and exclusion.

To explore wellbeing in this context, we employ Nordenfelt’s (21) theory of wellbeing, which defines it as the ability to realize one’s *vital goals* - the set of conditions necessary for an individual’s minimal happiness under *normal circumstances*. Yet for older migrants, these “normal circumstances” are often disrupted by the compounded challenges of aging and migration: language barriers, institutional exclusion, and the loss of culturally embedded support systems fracture the very conditions Nordenfelt presupposes as stable. Bourdieu’s (12) concept of social capital enriches this perspective by framing social networks and relationships as *strategic resources* that enable older adults to compensate for such disruptions and achieve their vital goals.

Drawing on Hall’s (22) conceptualization, culture is understood as operating through two interdependent systems: first, as a shared conceptual map that structures how people make sense of their world, and second, as language that enables communication and negotiation of these meanings. This dynamic framework of values, norms, practices and worldviews fundamentally shapes how individuals interpret their experiences and interact with others in social relationships. In this study, first language was used as a proxy for cultural identity, as it, though not a perfect cultural surrogate, often reflects deeply rooted social and communicative frameworks formed through early-life experiences (23). As older foreign-born individuals age in a new sociocultural context, their cultural background may continue to inform how they seek connection and interpret social roles in the host society.

Existing research on older adults’ social networks across diverse national contexts has established how demographic factors, socioeconomic status, and ethnocultural background shape the structure, quality, and function of social ties (24–27). In Sweden, studies of older immigrants have provided important but narrowly focused insights - for example, MacInnes et al. (28) on Finnish and Western Balkan migrants’ use of informal neighborhood spaces, or Magnusson and Kiwi (29) on Iranians’ reliance on ethnic and religious associations for integration. Johansson et al. (30) further highlights how non-European older migrants exercise agency in pursuing “good aging” through a combination of kinship, community solidarity, and Sweden’s welfare infrastructure. Yet these studies, while valuable, tend to isolate specific ethnic groups or focus on static network characteristics rather than the dynamic processes linking migration and aging to social connectedness. This study advances the field by examining how and why the intersection of migration and aging reshapes social ties and wellbeing across a broader spectrum of older foreign-born populations in Sweden.

The aim of this study is to investigate how older foreign-born adults in Sweden experience and navigate social connectedness as a determinant of wellbeing, addressing three key questions:

- How do perceived gaps between relational aspirations and actual social networks influence their wellbeing?
- In what ways do cultural backgrounds and migration experiences shape their sense of belonging in both private and institutional settings?
- What strategies do they employ to maintain wellbeing while negotiating identity and belonging in a new cultural context?

## Materials and methods

### Study design and setting

The research design for the study is based on the Glaserian paradigm of Grounded Theory (31), which emphasizes a systematic, iterative process of data collection and analysis. Key elements include theoretical sampling (selecting data based on emerging concepts), constant comparison (comparing data to refine categories), and theoretical saturation (stopping when no new insights arise). The research setting was a medium-sized municipality in southeastern Sweden with nearly 30% foreign-born population but otherwise a demographic composition that was representative of the average of the country.

### Participants

The study utilized qualitative data comprising five focus group discussions and one individual interview, conducted between October 2024 and February 2025. Participants were foreign-born individuals aged 60 years or older who met the inclusion criterion of being capable of engaging in conversations independently. To ensure representation across key dimensions such as immigration history, socioeconomic status and cultural background, several sampling strategies were employed. While we aimed to balance all these factors, primary sampling considerations focused on variations in age and migration-related characteristics - specifically country of birth, length of residence in Sweden, and age at immigration. These were prioritized due to their anticipated relevance to the study’s objectives.

The study used participants’ first languages as a proxy for cultural differences, organizing focus groups into one Chinese-speaking, one Finnish-speaking, two Arabic-speaking, and one Spanish-speaking group. This selection served a dual purpose: (1) representing the demographic composition of Sweden’s older foreign-born population, where Finnish and Arabic speakers are among the largest groups, and (2) capturing cultural dimensions aligned with the World Cultural Map (32). While Finnish, Arabic, and Spanish speakers reflect major immigrant communities in both Sweden and the study municipality, Chinese speakers - despite their smaller local and national presence - were included to provide linguistic and cultural contrast beyond the Indo-European and Afro-Asiatic language families (33).

Following the Chinese focus group, we conducted an individual interview with an 80-year-old Chinese-speaking participant (C5) who opted out of the group session but agreed to a one-on-one format. Respecting the participant’s preference for comfort aligned with Glaser’s (31) “all is data” principle, and we anticipated that this approach might enrich the dataset by capturing a deeper, uninterrupted perspective distinct from group dynamics. Preliminary analysis, however, revealed minimal variation attributable to age differences, leading us to prioritize migration status as a more salient factor in subsequent stages.

Recognizing substantial heterogeneity in immigration backgrounds and socioeconomic status within Sweden’s Arabic-speaking population, we strategically divided participants into two distinct groups: Swedish-speaking and non-Swedish-speaking, reflecting differing levels of integration. After initial sessions, we limited group sizes to 4–6 participants to ensure equitable speaking opportunities - a critical consideration given many participants’ limited Swedish proficiency. While traditional focus groups typically consist of 6–8 participants (34, 35), smaller groups of 3 or 4 participants are methodologically justified for participants with specialized knowledge, hard-to-reach populations or sensitive topics (36–38). Given the language barriers and recruitment challenges inherent in studying older migrants, this study employed “mini-focus groups” (38, p.17) to prioritize meaningful interaction and participant comfort. Notably, one Chilean participant withdrew immediately prior to the session, resulting in a final Spanish-speaking group of three. While we debated recruiting an additional group to mitigate potential data limitations, practical constraints (e.g., time) precluded this adjustment.

Table 1 is a list of all the 23 participants. The list is arranged in the order of interviews.

**Table 1:** List of participants.

No.	Age/Gender/Marital status	Reason for immigration	Country of origin	Age at the time of migration	Years lived in Sweden	Years of formal education	Employment status
C1	69/Female/Divorced	Family	China	31	38	16	Working
C2	63/Female/Married	Work	China	23	40	12	Working
C3	62/Female/Married	Family	China	25	37	16	Working
C4	66/Female/Married	Family	China	48	18	12	Pensioner
C5	80/Female/Married	Family	China	35	45	16	Pensioner
A1	62/Male/Married	Asylum	Iraq	45	17	24	Working
A2	62/Male/Married	Asylum	Iraq	45	17	16	Working
A3	62/Female/Married	Asylum	Iraq	46	16	20	Working
A4	64/Female/Widow	Asylum/Family	Syria	19	45	11	Working
A5	60/Female/Married	Asylum/Family	Turkey	13	46	13	Working
A6	60/Male/Married	Asylum	Iraq	37	23	13	Working
F1	62/Female/Divorced	Family	Finland	6	56	11	Working
F2	85/Female/Married	Work	Finland	30	54	9	Pensioner
F3	85/Male/Married	Work	Finland	30	54	6	Pensioner
F4	69/Female/Divorced	Family	Finland	19	50	10	Pensioner
F5	78/Female/Single	Family	Finland	29	49	8	Pensioner
A7	64/Female/Married	Asylum	Iraq	45	19	9	Pensioner
A8	68/Female/Widow	Asylum	Iraq	60	8	15	Pensioner
A9	64/Male/Married	Asylum	Iraq	47	17	16	Working
A10	67/Male/Married	Asylum	Iraq	49	18	18	Pensioner
S1	67/Female/Divorced	Asylum/Family	Chile	24	43	12	Pensioner
S2	76/Male/Divorced	Asylum	Chile	39	37	12	Pensioner
S3	68/Female/Married	Asylum/Family	Chile	22	46	15	Pensioner

### Recruitment of participants

Participant recruitment employed a multi-pronged approach tailored to availability and context. The first author visited various meeting places frequented by older adults, including eight municipal social centers designed to promote activities and social engagement among older individuals and people with disabilities. Notably, while these centers were distributed across major city neighborhoods—including several located in areas where approximately 80% of residents were foreign-born—we observed almost no attendance by older non-European immigrants at any of these locations. This observation sparked our interest in understanding the reluctance of older immigrants to participate in social activities with native Swedish peers. While we initially sought to recruit diverse participants for focus groups, we encountered significant challenges in encouraging them to either engage in conversations with strangers or travel to unfamiliar locations, ultimately necessitating alternative recruitment strategies.

As a result, we pivoted to collaborating with three local cultural organizations serving immigrant communities: a Finnish social center, a multicultural association, and a Syrian Orthodox church. After interviewing representatives from each organization, we requested their assistance in disseminating study information to potentially eligible members. This approach successfully enabled recruitment for both the Finnish and Arabic-speaking focus groups.

We acknowledge that Syrian Orthodox church members represent only one segment of the Arabic-speaking cultural spectrum, and that Finnish social center attendees may reflect particular social network preferences. This recruitment strategy inherently involved trade-offs between sample diversity and participant comfort—a deliberate choice prioritizing participants’ ease of engagement in the research process.

Participants in the Chinese-speaking and Spanish-speaking focus groups were recruited through a combination of convenience sampling (39) and subsequent snowball (network) sampling (39).

### Data collection

One general question was asked at all our focus group discussions: “*who do you turn to if you need someone to talk to or if you need some assistance*?” The discussion guide explored four key themes: (1) the composition and supportive functions of participants’ social networks, (2) the role of social relationships in health and wellbeing during aging, (3) how migration backgrounds shaped social connectedness, and (4) experiences with Swedish authorities (particularly healthcare). The same guide was adapted for use in the individual interview with the Chinese-speaking participant. The length of focus group discussion sessions and the individual interview was between 70 and 90 min. At the end of each session the participants were always asked to answer a structured questionnaire about their personal data: name, age, country of birth, years lived in Sweden, age at the time of immigration to Sweden, motive for migration, marital status, years of formal education, and employment status. All focus group discussions and the individual interview were conducted by the first author, with one session including another researcher from the team participating digitally as an observer. Discussions with the Chinese-speaking participants were conducted in Mandarin, the first language for the first author, also an authorized and active Mandarin-Swedish interpreter. The remaining focus group discussions were conducted in Swedish. A registered interpreter by the Swedish Kammarkollegiet was employed to the second Arabian focus group discussion. All interviews were audio-recorded and transcribed verbatim. A field research memo was written after each session, to document the researcher’s impressions of the participants, group dynamics, participant interactions, situations during discussions, and reflections on the primary findings and strategies for future recruitment of new focus groups.

### Member checking

Member checking was conducted by returning all transcriptions to participants for accuracy verification and resonance with their experiences. All participants approved the final transcriptions.

### Ethics approval and consent to participate

The study was approved by the Swedish Ethical Review Authority (Dnr: 2023-04950-01). Codes have been used to protect the participants’ anonymity. Both oral and written information about the study and consent forms were provided when contacting potential participants. Data collection began only after participants have given their verbal and written consent. Information about the study and consent were reiterated for all participants before each session. No data on political opinions or religious beliefs were collected.

### Data analysis

All data were analyzed using grounded theory methodology (31). The analysis proceeded in two phases: First, each session was coded individually; subsequently, constant comparative analysis was applied across all transcripts. The Chinese-speaking participant’s individual interview underwent initial separate analysis before being systematically compared with the Chinese-speaking focus group data. Upon confirming thematic consistency between these datasets (with no significant categorical discrepancies observed), the individual interview data were incorporated into the focus group analysis. The data from the sessions, along with the research memos, were examined line-by-line, with open coding applied to capture the informants’ perceptions and experiences. The data was conceptualized and coded into categories. Initial concepts and categories included for example “deep understanding,” “sense of belonging,” “recognition,” “identity and identification,” “acknowledgment vs. invalidation,” “reflection and revaluation,” and “acceptance and reconciliation.”

After analyzing the first four focus groups with Chinese speakers, the first Arabic-speakers, and the Finnish speakers, the core category “a quest for understanding” emerged, supported by subcategories “wanting to be understood,” “wanting to understand,” and “reaching for reconciliation.” To determine whether these findings were consistent among Arabic-speaking communities in Sweden, we conducted a second focus group with participants who had relatively shorter residency periods and varying levels of Swedish proficiency. Notably, their experiences aligned with the existing categories, further reinforcing the emerging patterns.

Finally, theoretical sampling (31) was used to recruit the Spanish-speaking focus group. This approach aimed to collect additional data related to emerging concepts, identify gaps, and refine the theoretical model. The new insights enhanced the depth and robustness of the core category and subcategories, confirming that theoretical saturation (31) had been achieved.

A new research memo was developed to synthesize and analyze the relationships between all emerging concepts and categories. Once the framework of hypotheses stabilized, we systematically reviewed relevant literature, including theories and policy documents on health and wellbeing, culture, social capital, and research on social networks among older migrants. Using emergent fit (40), we incorporated components of pre-existing theories that aligned with our data, treating them as new evidence for comparison with the emerging hypotheses. This process preceded the integration of final conclusions.

Following each interview, the research team collectively analyzed the generated data, including interview transcripts and research memos. To ensure the trustworthiness of the findings, we conducted peer-debriefing sessions with the first author, who served as the primary coder (41). Additionally, we systematically sought disconfirming evidence (42) to validate that our findings accurately represented both individual interpretations and the shared perspectives on the issues examined.

## Results

The participants chose to engage in meaningful social relationships that provided a context in which they could best understand others and be understood by others. This was because they perceived the essence of social interaction as an opportunity to explore, express, and affirm their thoughts, feelings, and experiences. This manifested as a core phenomenon - *a quest for understanding* - comprising three interrelated dimensions:

- *Wanting to be understood*, reflecting their need for recognition, inclusion, and appreciation of their unique experiences, cultural identities and personal histories;
- *Wanting to understand*, demonstrating their reflections on both shared challenges and distinct cultural strengths when they navigated their identities and integration into Swedish society; and
- *Reaching for reconciliation*, representing their ultimate pursuit of both self-acceptance and acceptance within their social world.

These findings revealed the participants’ dual needs for validation through being understood and connection through understanding others. The two primary desires proved complementary rather than contradictory: as participants developed empathy for others’ perspectives, they simultaneously created conditions for reciprocal understanding of their own experiences. Over time, this process evolved into a deeper reconciliation of cultural, relational, and generational divides, enabling participants to negotiate their evolving identities while seeking common ground, as they tried to adapt to the social world they inhabited in.

In the following presentation, the first two subcategories (*wanting to be understood* and *wanting to understand*) are structured according to focus group discussions, as notable homogeneity within groups—and divergence between them—arose from varying cultural distances from Swedish society and differing migration backgrounds. In contrast, the final subcategory (*reaching for reconciliation*) is presented from individual perspectives, as participants across all groups sought to depict their social lives in a holistic, nuanced manner, with minimal intergroup variation.

### Wanting to be understood

The findings revealed that participants predominantly sought social connections with compatriots who shared their first language and life experiences, while facing varying degrees of difficulty building relationships with native Swedes. These challenges stemmed from language barriers, cultural distance, the reserved nature of Swedish social interactions, and societal prejudices—particularly toward Arabic-speaking communities. Participants from these groups, including long-term residents who identified as both Swedish and culturally hybrid, reported being stereotyped or perceived as “outsiders” despite their integration efforts.

The Finnish-speaking participants were unique in reporting that they did not perceive themselves—nor were they perceived—as immigrants. Nevertheless, they valued gatherings with fellow Finns, where they could speak Finnish and engage in shared cultural practices such as Finnish music, art, cuisine, and handicrafts. They usually celebrated all the traditional Finnish holidays together. Notably, they emphasized cultural distinctions between Finns and Swedes, attributing these differences to Finland’s historical experiences of war and poverty, which shaped a distinct national mindset. Some referenced the prejudices faced by earlier Finnish immigrants in Sweden during the 1950s–1970s, many of whom had migrated due to post-war hardships. A lingering “*little brother versus big brother*” dynamic with Sweden also persisted.

F3: *“We’ve lived in Sweden for a very long time. We’re really happy here - there’s nothing missing. But we always vacationed in Finland when we were working and in the first years of our retirement.”*

F2: *“I enjoy living in the countryside, and I like being here. I feel comfortable when everyone speaks Finnish. I’ve only learned a little Swedish.”*

F4: *“This place actually means quite a lot. It’s the language and the sense of community. For those who are alone, this means something extra. Personally, I’ve served on the board of the Swedish Rheumatism Association and coached for the local swimming club. So I have both. I’m on both sides. And it’s really fun that I get to do that.”*

Like the Finns, the Spanish-speaking participants reported good integration after nearly five decades in Sweden, with Swedish friends, colleagues, neighbors, and even family members. Yet, they still identified themselves as immigrants, citing two key factors. One was language barriers: Their self-perceived imperfect Swedish affected how seriously they felt they were taken. Another was cultural differences in sociality: They described Latin American social interaction as more expressive, with music and dance as intrinsic to identity and emotional expression.

S3: *“My husband, who is fully Swedish, spends much more time with us than with Swedes. He thinks it’s very pleasant. There’s always lots of food, music, and dancing when we get together. Music is something in our genes - I’ve been playing since childhood.”*

S2: *“My daughter is married to a Swede. She’s kind and always invites me to their place for Christmas. But I feel like it’s just ‘how are you?’ and ‘fine, thanks!’ It’s a bit dull at the Swedes’ place. They do not speak Spanish. I might as well stay home and listen to my old cassettes.”*

S3: *“But after all, I think we Chileans have managed pretty well in Sweden, do not you agree?”*

S2: *“Absolutely! I’ve had lots of friends over the years - Swedes, Finns, immigrants from all over.”*

S1: *“I think it depends a bit on where you end up - what workplaces, what social circles, what environments.*

The local Chinese community was relatively small compared to other immigrant groups and lacked organization. The Chinese-speaking participants described pronounced social isolation, struggling to form meaningful connections with either Swedes or fellow Chinese. They identified greater linguistic and cultural distances than other immigrant groups, with Swedish cuisine posing a particularly symbolic challenge—framed not just as dietary discomfort but as a cultural and existential longing for familiarity. They emphasized that only Chinese peers of similar age and life experiences could fully understand their perspectives. Additionally, they noted a lack of cohesion within the Chinese community, citing mutual distrust and lower tolerance among compatriots compared to other immigrant groups.

C2: *“Of course it’s important to have friends! But it’s so difficult with Chinese people.”*

C4: *“Chinese people are the worst at sticking together.”*

C3: *“I agree.”*

C1: *“Yes, say what you will about those from the Middle East and Africa - they really stand up for their countrymen like brothers and sisters when it matters, for real! It makes you envious. We Chinese are far too lacking in this regard.”*

Moderator: *“But I asked about your social relationships and networks, and so far you have only talked about Chinese people you have met in Sweden. Should I interpret this as meaning you think relationships with other compatriots in your environment are important?”*

C1, C2, C3, C4: *“Of course it’s important!!”*

Moderator: *“Why is that?”*

C3: *“It’s that depth of understanding. My Swedish husband really wants to understand me - I have a kind husband - but sometimes it’s hard to make him understand when I want to explain more complicated thoughts and feelings. There’s a little empty room that’s hard to fill. He cannot feel what I feel.”*

C2: *“It might be about language. Maybe you have difficulty fully conveying what you feel. You meet halfway.”*

C1: *“It could also be about life experiences. It’s so much easier to understand each other if you have lived in the same country during the same time periods.”*

C3: *“Then there’s food too. Food is important.”*

C1: *“Food is a big deal. In this regard, we Chinese are truly different. We stand out in the world - maybe we are only similar to a few other East Asian countries. Actually, Middle Eastern food is not all that different from Swedish food. They can appreciate each other’s cuisine. Even African food is closer to European food.”*

In contrast, Arabic-speaking participants expressed strong solidarity with compatriots and other Middle Eastern communities, rooted in shared language, history, and social customs. Many cited the church as a central hub for cultural preservation, offering activities for all ages—from children and working-age adults to older pensioners. These included traditional festivals, language classes, handicrafts, and charity work. The congregation also organized separate activities tailored for women and men, fostering a sense of community across generations and genders. At the same time, they reported a sense of resignation when it came to integrating into Swedish society, describing persistent barriers to forming friendships with Swedes and structural challenges like employment discrimination.

A7: *“When you come here alone, you really need someone you know you can turn to. I’m grateful for my congregation - I’m here often. I have several close friends who also belong to this church community.”*

A8: *“Not long ago, I had surgery. When I recovered enough to go out, my first destination was coming here to the congregation.”*

A10: *“Swedes aren’t really into community. I find them a bit difficult and dull. When I first came to Sweden at 48, I wanted to integrate into Swedish society - but I could not. I could not break into the job market. Socially, though, we have no shortage of community among ourselves. We have our immediate families, who meet daily or weekly; then extended relatives like cousins we spend time with; and beyond that, there’s our congregation - lots of compatriots or people from the same region who meet once, twice, or three times a month. We throw parties when we gather. Our families also visit each other often.”*

Even those who immigrated young and spent most of their lives in Sweden shared these frustrations, feeling misunderstood and undervalued due to stereotypes and non-recognition of their qualifications.

A4: *“I came to Sweden long ago, and I’ve worked here my whole life. Yet I still do not have many Swedish friends - and it’s not because of the language. Once, I asked a neighbor who was my age if we could spend time together. But during our walks, all her questions were about money: ‘How could you, a widow with kids, afford a house? Where did the money come from?’ Finally, I told her: ‘My husband worked himself to death here. We pay taxes, just like you. We’ve done right by this society.’”*

A5: *“We’ve lived in this country for a long time. We’ve worked, paid taxes, and done our part - so we have the right to speak up about what we think. I’ve lived in this area the whole time, and it’s changed a lot. At first, I had many Swedish friends, but as people from all over the world moved in, most Swedes left. So no, we do not have much contact with Swedes anymore.”*

In conclusion, participants consistently found the deepest understanding among those who shared their language and life experiences, reinforcing their reliance on ethnic communities. While Finnish and Spanish speakers felt accepted (if not fully understood) by Swedish society, Arabic and Chinese speakers reported more systemic exclusion. The former derived strength from tight-knit communities, whereas the latter grappled with a broader sense of displacement.

### Wanting to understand

The question of social relationships prompted participants to reflect deeply on their lived experiences, sparking moments of introspection. This desire to understand extended beyond personal connections to include their aspirations to comprehend both Swedish society and their heritage cultures, which continued to shape their identities in profound ways.

Arabic-speaking participants observed that immigrant segregation from native Swedes had intensified over time, particularly for Middle Eastern communities. They attributed this to four interrelated factors. One was language barriers: While acknowledging labor market discrimination, they noted their children—who spoke fluent Swedish—integrated more easily, suggesting age of arrival and language proficiency were pivotal to social acceptance. Another was cultural distance: They reinterpreted Swedish reserve as a cultural norm rather than exclusion, observing similar behavior among Swedes with family and friends. The third was negative stereotypes: They critically reflected that some immigrant groups’ misconduct (e.g., youth gang crime) exacerbated prejudices affecting all immigrants. The last reason was policy failures: They found political efforts appeared more focused on maintaining public approval than addressing systemic social issues. Despite these challenges, they expressed strong appreciation for Sweden’s rule of law, welfare system, and governance, viewing it as their permanent home. To bridge understanding, their community organizations regularly hosted Swedish officials (e.g., police, healthcare workers) for dialogues on societal systems.

A8: *“If you compare Sweden with many other countries, we have it much better here.”*

A7: *“There are many positive aspects of Swedish society. Everyone is pleasant - at the healthcare center, the Social Insurance Agency, really everywhere when dealing with government offices. At the hospital, they do everything - they ask how we are feeling, what we need. When you arrive, they greet you; they thank you when they leave. They do everything. This is rare in other countries.”*

A10: *“We’ve learned many things. Above all, we have learned patience. You must queue. You must be on time. There are rules. Right must be right. The law is the law. Even though we are not fully accepted by the Swedes, we are ready to fight for this country - because this is our land now.”*

The Chinese-speaking participants identified a lack of institutional guidance as a major hurdle, particularly given Sweden’s complex legal and cultural differences from China. Their proposed solution was a community-led chat group, where established Chinese diaspora could share practical knowledge (e.g., navigating bureaucracy) to ease newcomers’ transition. They believed that only a Chinese peer - particularly one who had navigated the transition between Chinese and Swedish contexts - could effectively identify which aspects of Swedish society might prove most surprising, confusing, or institutionally critical for newcomers.

C4: *“As someone who does not speak Swedish, I enthusiastically support having a functioning local Chinese network! Even just a chat group goes a long way. Whenever I have a question, someone in the group who knows will answer. There’s so much bureaucracy for everything in Sweden.”*

C1: *“It’s good we can help each other. There’s a real need for this.”*

C2: *“I think we should also meet sometimes and get to know each other better, do things together if people want. Those who are better off could contribute a little more to those struggling. But everyone must contribute. It needs to be a mutual, balanced, and fair relationship.”*

In contrast, discussions among Finnish- and Spanish-speaking participants focused less on integration struggles and more on preserving their heritage while enjoying bicultural fluency.

The Chileans nostalgically recalled the golden 1980s, when thousands of immigrants from Chile and other Latin American countries arrived in this city, when cross-cultural activities (playing football, boxing, dancing) fostered solidarity with the locals. Back then, everyone was friends. Though less active now, their community remained tightly knit, mobilizing for crises for their compatriots in Chile and in Sweden. They established a local Spanish-language radio station, sustained through collective volunteer efforts, which had become their digital gathering place. They broadcasted news and music from Latin America, sometimes even receiving calls from Chile. The radio station served as an acoustic bridge, rendering their distant homeland simultaneously remote and proximate - geographically far yet culturally immediate.

One of the participants, S2, had worked for the radio as a volunteer for many years.

S2: *“I do it because on the radio, I can do so much of what I love. People call in, and we discuss music, travels, anything at all. It’s a lovely way to socialize. I used to host my own show called The Corner.”*

S1: *“The Romantic Corner! He played beautiful romantic music!”*

S2 (laughing): *“Yes, gorgeous romantic songs from around the world! But then people started complaining that I played Swedish and English tracks too. They only wanted Spanish music!”*

S3: *“This radio station has become vital for us Chileans in town. I even feel a bit empty when I’m traveling and cannot tune in. We do not meet as often anymore, but if something happens back home or a fellow Chilean faces hardship, we always hear the news on the radio and rally to collect funds. We’re still here when it matters.”*

The Finnish speakers viewed themselves as custodians of a diminishing cultural heritage in Sweden, particularly as new Finnish migration had ceased. This perception persisted despite Finnish Swedes’ status as a recognized national minority in Sweden - a designation that guarantees official protection and preservation of the Finnish language and culture. The participants gathered weekly simply to speak Finnish, underscoring the language’s vital role in fostering a sense of unity and belonging within the community. They emphasized the psychological toll of aging in a non-native language. Struggling to express complex emotions, memories, and thoughts in a second language could be deeply frustrating and stressful, particularly for those who never had the chance to become fluent. The added challenge of potential dementia in old age might further complicate their situation. This was why many desired to move into a Finnish-focused nursing home with Finnish-speaking staff when they could no longer manage on their own.

F1: *“I work at a nursing home with a Finnish profile - meaning we incorporate both Finnish and Swedish cultures. We have two dementia units. Language is crucial here. We have staff who speak Finnish, because often when dementia progresses, residents lose their Swedish language skills. When it’s someone like my mother, who’s experiencing early memory loss, having caregivers who speak Finnish makes all the difference. It lets her age with dignity.”*

Moderator: “*So dignity is what’s at stake here?”*

F1: *“Exactly – it’s about being truly understood.”*

F4: *“It’s about being truly seen. I myself will turn 70 next year. In 10 years, I’ll be 80. It’s approaching. I’m so grateful that Finnish-speaking nursing homes exist here. The best thing is that they embrace both Swedish and Finnish languages and cultures, though as the years pass, I suspect I’ll need Finnish more and more. My son and I are close, but we have lost our Finnish connection. He spoke it as a child, but now. it’s gone.”*

F5: *“I just hope there will still be spots available for us when the time comes - those Finnish nursing homes!”*

In conclusion, with thoughtful reflections, the participants tried to navigate dual cultural worlds with both intellectual curiosity and profound respect - a perception stemmed from their fundamental need to comprehend both their adopted social realities and their transforming selves.

### Reaching for reconciliation

The participants were consciously negotiating between their aspirational wants and needs (cultural preservation, meaningful connection, generational continuity) and their actual living circumstances (integration challenges, cultural gaps, evolving identities). This process manifested as they reflected on their life journeys, developing nuanced understandings of social relationships that balanced cultural negotiation (preserving heritage while adapting to Swedish society), relational equilibrium (needing connection yet valuing independence), and generational acceptance (releasing expectations while finding new meaning in their descendants’ integration). The term “reconciliation,” rather than mere “acceptance,” emphasizes the active deliberation behind this stance - participants did not passively surrender to circumstances but engaged in continuous meaning-making to align their ideals with reality.

While some participants acknowledged the challenges of integrating into Swedish society, most had negotiated a balance between their dual identities as immigrants and long-term residents. This delicate equilibrium was perhaps best illustrated by one participant’s story (A7, smiling): *“My Swedish neighbor and I had a little ritual for years. I’d bring her freshly baked bread - she’d take it with a quick ‘tack!’ through a barely opened door, never inviting me in. But that was our dance, you know? She kept her ways, I kept mine, and somehow that worked just fine.”* Her reflection resonated with others in the group, including A9’s pragmatic observation: *“This is the way they are, and that is the way we are - it’s simply how things are, and we must learn to live with it.”* Together, these accounts reveal a quiet recognition that full assimilation into Swedish norms - or full acceptance as equals - might remain elusive. Yet within that space, participants cultivated a hard-won mutual understanding, choosing respect over resistance while preserving their own cultural rhythms. This sentiment was echoed by another participant, who simply stated, *“People are just different, and that is alright”* (S3), encapsulating a pragmatic acceptance of diversity without the expectation of complete integration. This acceptance of cultural differences allowed them to navigate their lives with a sense of peace. They valued the trust they placed in Swedish institutions and the stability Sweden providing, often describing it as the best place to live despite the social and cultural barriers they faced in their personal contacts with native Swedes. Even though some had considered moving back to their home countries after retirement for a more comfortable aging - with more favorable food, climate, cultural activities and social community - most participants now regarded Sweden as their home, not only because of the lives they had built here but also because of their children and grandchildren. After all, “*home is where my children and grandchildren are*,” as several participants poignantly stated.

One point of consensus among participants across all groups was the growing cultural gap between themselves and their younger descendants, as one participant starkly observed:*” The best scenario is to grow old surrounded by family. But my daughter has already told me quite plainly: ‘I see how you care for your mother, but I will not be able to take care of you the same way you have cared for her’”* (C5). Older generations emphasized the importance of family unity, respect for traditions, and, most notably, the expectation that their children would take responsibility for them in old age. However, this expectation was not merely about practical support - such as providing physical care or financial assistance - but also about emotional solidarity and a sense of enduring familial devotion. For many, true care meant not just doing for one’s parents but caring about them - maintaining close bonds, showing affection, and ensuring that older people felt valued rather than abandoned. This deeper, affective dimension of care was often what participants felt was most at risk as younger generations assimilated into individualistic norms. This disconnection fosters some frustration and a sense of loss among the participants. “*I cannot force them to like my language and my culture,*” one participant (S2) remarked, encapsulating the bittersweet acceptance of this reality. At the same time, they also acknowledged that their children and grandchildren had become a bridge to Swedish society, offering them opportunities to learn about and integrate into the culture more deeply than they might have otherwise. Despite their own challenges and struggles, they found comfort in knowing that their children had achieved a level of integration they themselves could not, viewing it as a testament to their sacrifices and efforts. This reconciliation allowed them to let go of the expectation that their cultural legacy would be fully embraced by their descendants.

For many participants, aloneness was not merely a state of isolation but a conscious pursuit with two distinct yet intertwined purposes. First, it served as a retreat from social interactions perceived as hollow or draining - a way to preserve dignity and emotional wellbeing by rejecting the superficial. As one participant succinctly put it, *“Sometimes, being alone is better than being in bad company”* (C5). This sentiment was especially prominent among Chinese participants, who often framed solitude as a boundary against unsatisfying relationships, prioritizing quality over quantity in their social lives. Yet solitude also held a more profound, affirmative role: a space for self-discovery and a deepened relationship with oneself. Freed from the noise of obligatory socialization, participants engaged in introspection, creative hobbies, or immersive experiences in nature. “*I find everything I need in the forest,”* shared one Finnish participant living in a rural area, *“I feel a connection with nature and the animals that is more fulfilling than many human relationships”* (F3). Here, solitude transcended mere withdrawal; it became a way to engage more deeply with life itself - to observe, reflect, and ultimately redefine what meaning and connection could entail.

A clear consensus emerged among participants: the ideal social relationship was one of balance. The first balance was between autonomy and connection - individuals should maintain a strong sense of self while engaging with others. The second involved self-sufficiency and interdependence: they valued the ability to manage daily life independently yet remain open to leaning on others when deeper connection was needed. Beyond these dualities, many aspired to a vision of belonging that felt both personal and universal - one that transcended labels like “immigrant” and embraced a shared identity as human beings. As one participant poignantly remarked, *“I am friends with everyone, and everyone is my friend”* (S1).

## Discussion

This study illuminates how older migrants in Sweden navigate belonging through a *quest for understanding* - a struggle to align vital social goals with lived realities. Below, we discuss these dynamics through three research lenses: relational gaps, cultural scripts, and agentic resilience.

### Relational gaps and divergent wellbeing outcomes

Consistent with Nordenfelt’s theory of wellbeing as goal-reality alignment (22), participants’ relational gaps - the disparity between desired and actual social connections - varied significantly by cultural-linguistic background, with profound implications for wellbeing.

- Finnish speakers’ minimal gaps reflected their intersectional advantages: national minority status, geographic proximity, and decades of community advocacy that mitigated early discrimination. Their bilingual fluency enabled access to both Finnish and Swedish social worlds, allowing them to preserve cultural identity while integrating structurally (e.g., Finnish nursing homes).
- Arabic speakers faced systemic marginalization despite strong intra-group solidarity. While their tight-knit communities provided emotional support, exclusion from Swedish circles persisted due to language barriers and cultural stereotypes. Even long-term residents who self-identified as Swedish reported being perceived as outsiders, highlighting how prejudices compounded relational gaps.
- Chinese speakers endured the largest gaps, with neither robust ethnic networks nor Swedish inclusion. Cultural distance (e.g., food as symbolic displacement) and mistrust among compatriots left them doubly isolated. Their struggles reflected a scarcity of social resources - linguistic, institutional, and communal- to bridge aspirations and reality.

These disparities align with Bourdieu’s social capital theory (13): Finnish-speaking participants’ cultural/linguistic capital facilitated access to Swedish networks, while Arabic and Chinese speakers’ exclusion perpetuated inequality, limiting their ability to convert social ties into wellbeing.

### Cultural scripts and belonging in private vs. institutional spaces

Arabic-speaking participants expressed profound appreciation for Sweden’s public systems - healthcare, rule of law, and welfare. Such trust anchored their belonging at a structural level, with many declaring Sweden their permanent home. Yet this official belonging contrasted sharply with the social exclusion they experienced in daily life. This duality aligns with Ager and Strang’s (43) framework, where institutional integration (e.g., access to services) can occur alongside persistent social and cultural barriers. In contrast, Chinese participants lacked even this institutional foothold, citing opaque bureaucracy and minimal guidance. Their isolation was thus doubly compounded: excluded from both Swedish social circles and institutional support networks.

Hall (23) argues that culture provides mental “maps” through which people decode their experiences - what is familiar becomes “home,” while the unfamiliar is marked as “other.” These conceptual maps clarify why participants trusted Swedish institutions yet felt socially excluded: their cultural frameworks rendered them “legible” to systems but “illegible” in everyday Swedish life, a paradox in the multicultural Swedish society documented by Ålund and Schierup (44). This misalignment underscores that belonging requires not just structural inclusion but shared codes of understanding.

### Understanding as the foundation of belonging

At the heart of participants’ narratives lay a quest for mutual understanding - a desire not only to *be understood* as individuals of worth but to *understand* the cultural codes of their adopted homeland. This reciprocal process emerged as the bedrock of belonging: without Swedes’ *will to understand* their histories, hybrid identities, or emotional languages, structural inclusion (e.g., residency, healthcare access) remained hollow. Finnish speakers’ relative success in achieving belonging reflects both their long-term residence and Sweden’s eventual recognition of their community as a national minority- factors that mitigated initial post-war discrimination. While their geographic, linguistic, and cultural proximity provided foundations for mutual understanding, it was the accumulation of shared time and institutional validation that transformed tentative acceptance into durable belonging. In contrast, Arabic and Chinese speakers faced asymmetrical understanding - they invested heavily in comprehending Swedish norms, yet felt their own identities were dismissed as incomprehensible. This asymmetry bred a belonging limbo: officially welcomed by Sweden’s systems yet socially orphaned by its people. The findings indicate that belonging cannot be legislated into existence; it flourishes only where understanding is mutual, curious, and unconditional.

### Agentic resilience: negotiating identity and belonging

The study reveals how older migrants actively reconfigured their social worlds to sustain wellbeing amid gaps between aspirations and reality. Their strategies reflect *pragmatic resilience* - neither passive assimilation nor resistance, but a dynamic recalibration of identity and connection. Participants with linguistic/cultural capital leveraged dual identities to access resources while preserving heritage, or mediated between generations, with descendants facilitating Swedish connections. This aligns with Berry’s acculturation model (6), where integration (retaining heritage + adopting host culture) correlates with higher wellbeing. In contrast, participants with constrained fluidity cultivated alternative belonging by community solidarity. Arabic speakers transformed their church into a cultural hub, offering language classes and intergenerational activities. Chilean elders invented traditions through a volunteer-run radio station, broadcasting news and music in Spanish and mobilizing crises support. Chinese speakers accepted isolation but proposed self-help solutions (e.g., chat groups). All these created a parallel public sphere countering Swedish exclusion. Crucially, participants’ resilience strategies - from ethnic community-building to solitude - were not retreats from belonging but *workarounds* for understanding’s absence. Participants also recalibrated expectations to align with reality. While some Arabic speakers normalized Swedish reserve, diffusing resentment while acknowledging limits, some Chinese speakers attributed exclusion to perceived unbridgeable cultural distance, framing their language and traditions as uniquely opaque. While Arabic speakers externalized exclusion as a Swedish cultural trait, Chinese participants internalized it as a Chinese characteristic, reflecting divergent coping mechanisms under similar marginalization. Finally, many participants relinquished expectations of cultural transmission, instead valuing descendants’ integration as a legacy of sacrifice.

## Conclusion and implications

This study, grounded in Nordenfelt’s theory of wellbeing and Hall’s cultural frameworks, reveals that older migrants’ social belonging in Sweden hinges on mutual understanding - a reciprocal process often disrupted by asymmetries in cultural legibility. Our findings indicate that belonging is multilayered. Finnish speakers’ long-term residence and minority status facilitated structural and relational belonging, while Arabic and Chinese speakers faced belonging limbo - legible to systems but illegible in everyday interactions. Participants’ resilience strategies varied by cultural capital: while bicultural fluency (Finnish/Chilean) enabled dual belonging, insular solidarity (Arabic) countered exclusion through ethnic institutions, self-reliance (Chinese) emerged where other resources were scarce. These reflect Bourdieu’s social capital disparities, underscoring how structural barriers shape agency. Our findings advocate understanding as policy imperative. Ultimately, the study reframes aging migrants’ wellbeing as a negotiation of belonging - where success depends not just on migrants’ adaptability but also on Sweden’s willingness to see them fully.

### Methodological reflections

Reflexivity, a key strategy for ensuring credibility in qualitative research (45), was critical given the first author’s dual role as an immigrant and geriatric nurse. Her “insider” status may have facilitated recruitment and trust, enabling participants to share openly, but also risked shaping findings through personal preconceptions. Team discussions mitigated this by broadening interpretive perspectives.

### Limitations and future directions

While this study captures diverse experiences, its recruitment through cultural/religious organizations may bias the sample toward socially engaged individuals. Future research should include isolated populations and explore intersections of gender, class, and migration history. Longitudinal designs could track evolving wellbeing across immigrational contexts.

## Data Availability

The datasets presented in this article are not readily available because anonymized data might be shared on reasonable request. Requests to access the datasets should be directed to ingrid.hellstrom@mchs.se.
